# An Analysis of Outcomes Following a Central Line Associated Blood Stream Infections (CLABSI) Reduction Quality Improvement Project in a Tertiary Care Center

**DOI:** 10.7759/cureus.42501

**Published:** 2023-07-26

**Authors:** Ronald Harris, Morgan Rosser, Nitin Mehdiratta, Anand Chowdhury, Becky Smith, Vijay Krishnamoorthy

**Affiliations:** 1 Anesthesiology, Duke University School of Medicine, Durham, USA; 2 Anesthesiology, Duke University Medical Center, Durham, USA; 3 Pulmonary and Critical Care Medicine, Duke University Medical Center, Durham, USA; 4 Infectious Disease, Duke University Medical Center, Durham, USA

**Keywords:** central venous catheters, central line-associated infections (clabsi), intensive care unit, infection control, quality improvement

## Abstract

Central Line Associated Blood Stream Infections (CLABSI) continue to be a significant complication for hospitalized patients. Hospitals have used various strategies to reduce CLABSI events due to the significant complications and associated costs. In this QI analysis, we examined the impact of a CLABSI reduction quality improvement project within a single ICU at a tertiary care medical center. Absolute CLABSI counts were compared between this ICU and other health system ICUs that did not implement the bundle. A sustained reduction in absolute CLABSI counts to or near zero was observed over 17 months after implementation. ICUs not performing the interventions during this time consistently reported ≥ 2 CLABSI per month. Further analysis is needed to assess causality and the generalizability of bundle components to other ICUs. These findings may provide other health systems with additional strategies to prevent CLABSI and provide consistent, evidence-based supportive care to critically ill patients.

## Introduction

Central lines are critical to administering lifesaving therapeutics. However, they also offer a direct path for pathogens to enter the bloodstream and cause infection. Central Line Associated Blood Stream Infections (CLABSI) continue to be a significant complication for hospitalized patients. In the intensive care unit (ICU), the vast majority of patients require central venous access [1], placing them at risk for preventable hospital-acquired infections (HAI). CLABSI are known to contribute to several adverse outcomes, including increased morbidity and mortality, length of stay, and added mean healthcare expenditures upwards of $129,000 [2-4]. As such, hospitals have employed various strategies aimed at reducing the number of CLABSI over the past decade, including empowering nurses to stop procedures, additional training, attention to site selection, checklists, prompt removal of unnecessary catheters, and bundles, including these and other changes [5,6]. For individual units and hospitals, preventing occurrences of CLABSI remains an important task in providing excellent patient care and improving patient outcomes. We describe a systematic approach to reducing CLABSI in a single ICU within a tertiary care hospital through a quality improvement (QI) intervention and compare its impact on ICUs unexposed to the QI intervention.

## Materials and methods

Identifying contributors to CLABSI

In June 2021, our institution's surgical intensive care unit (SICU) implemented a quality improvement (QI) initiative to reduce CLABSI. This study was part of the Quality Improvement: Active Surveillance, Outbreak Investigations and Bundled Interventions to Prevent Healthcare-Associated Infections (HAI) study approved by the Duke University Health System Institutional Review Board (IRB: Pro00104110). While CLABSI rates were high across the health system, the SICU had the highest absolute CLABSI count from 2020-2021, thus prompting a detailed evaluation of contributing factors. A multidisciplinary group of stakeholders utilized an A3 process [7] to identify potential contributors to the high CLABSI rate. An A3 is an organizational and management tool that is used to understand, analyze, and correct a problem [8]. Involving stakeholders in a structured dialogue about a problem generates insights and solutions into a succinct framework that outlines responsibilities, next steps, and success metrics [8]. CLABSI was defined according to the National Healthcare Safety Network criteria as a primary bloodstream infection in a patient who had a central line in for greater than 2 hospital days and was not associated with another site of infection.

**Figure 1:**
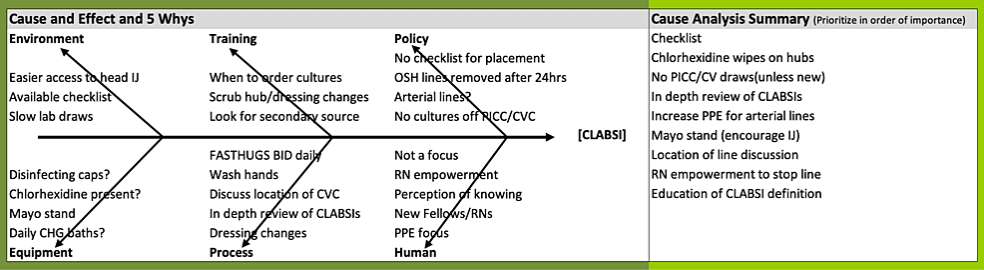
Fishbone Diagram Outlining Factors Contributing to CLABSI CLABSI: Central Line Associated Blood Stream Infections; CVC: central venous catheter; CHG: chlorhexidine gluconate; PICC: peripherally inserted central catheter

Following iterations through the A3 process by stakeholders, the main contributors to CLABSI included factors affecting sterility during insertion, proper maintenance of lines, and practices related to ordering blood cultures. These findings were organized and summarized with a fishbone diagram (Figure 1). Sterility was impacted when nurses did not feel empowered to speak up while witnessing contamination events. Particularly with new staff in the ICU (RNs, fellows, etc.), this likely resulted in lost opportunities for correction and teaching. Line maintenance contributed to CLABSI events when lines placed before being admitted to the SICU remained inserted for prolonged periods. Lines and catheters placed outside the ICU often remain inserted longer, increasing the risk of infection [9,10].

Additionally, central lines previously placed at outside hospitals may have a greater opportunity for contamination due to differing clinical protocols for line insertion and maintenance. In addition, a daily assessment of readiness for central line removal was not a consistent part of the discussion on daily rounds. Finally, the indications for ordering blood cultures varied widely in clinical practice without standardized guidelines.

Intervention bundle 

Continuing through the A3 process, a bundle of interventions was implemented in the SICU to reduce CLABSI. These interventions were rolled out over several months and addressed the gaps observed during line insertion, maintenance, removal, and infection prevention practices (Table 1). Education reinforcement for line maintenance and procedures and a new rounding mnemonic was implemented in July 2021. New chlorohexidine-alcohol (CHG) swabs and the procedure checklist were added in August 2021. A new blood culture algorithm was added at the end of January 2022. The A3 process was a consistent framework for continuous evaluation during each successive project addition.

**Table 1:** Summary of CLABSI Reduction Strategies CLABSI: Central Line Associated Blood Stream Infections; CHG: chlorhexidine gluconate

Main Factor	Intervention
Insertion	Nurses empowered to stop procedures
	New procedure checklist
	New larger CHG swabs
Line Maintenance	Review & Documentation (line indication, CHG bathing, dressing/tubing changes, hand hygiene, device and tube labeling
Removal	Rounding Checklist
	Guidelines on line removal & rewiring
Infection Prevention	Prohibit blood draws via central lines
	New blood culture algorithm

Insertion

To decrease the risk of introducing an infection during line insertion, a new procedure checklist was implemented for staff to follow before and during the procedure. A time-out was reinforced before starting, and nurses were encouraged and empowered to stop the procedure if they observed an instance of contamination. New larger CHG swabs were utilized for sterilizing the skin prior to insertion. The observing and/or supervising staff member completed the checklist, attesting that proper protocols were followed and whether there were any concerns. Checklists were then placed at a physician's office to allow for the creation of a CLABSI insertion record. This record allowed for case review, review of potential safety events, and continuous improvement of processes.

Line Maintenance

To improve the care of the central line once it was placed, interventions aimed at line maintenance were part of the CLABSI reduction bundle. Improved line maintenance centered around proper documentation and practices related to daily line care. Daily checklists for line inspection and dressing changes and proper dating of IV tubing and dressings were added in collaboration with critical care nursing colleagues. An emphasis was placed on scrubbing lines and hubs before and after any manipulation and inspecting lines daily. Dressings were changed after 48hrs of gauze and 7 days for non-gauze. Lastly, routine blood draws from central lines were discouraged; in patients with challenging access, blood draws from a central line (for laboratory analyses) could only be performed if an order from a provider in the EHR was placed.

Removal

An important piece of CLABSI reduction was the daily consideration of removing the central venous catheter. This adheres to the mantra: "A CLABSI is impossible if the patient does not have a central line." Greater scrutiny was placed on assessing the need for lines daily and removing unnecessary lines as soon as possible. Rounds were standardized to include indwelling catheters "I" as a part of the rounding mnemonic FAST HUGS BID (Feeding, Analgesia, Sedation, Thromboprophylaxis, Head of bed elevated, Ulcer prophylaxis, Glycemic control, Spontaneous breathing trial, Bowel care, Indwelling catheter, Drug de-escalation) [11, 12]. Lines placed at outside hospitals were removed within 48 hours following admission.

Infection Prevention

A number of new infection control practices were added to reduce CLABSI. In addition to discouraging the collection of routine labs from central lines, it was discouraged to rewire central lines unless absolutely necessary. Temporary hemodialysis lines were changed to tunneled lines in patients with prolonged kidney injury and a low likelihood for expedient recovery. Lastly, a new blood culture algorithm was implemented to refine the indications for ordering blood cultures and improve stewardship.

## Results

Utilizing data from performance services, absolute CLABSI counts within the SICU and all other health system ICUs were analyzed before and after the CLABSI QI intervention. Intervention elements were rolled out staggered throughout 2021 and into 2022. In the year prior to the intervention, the most common pathogens associated with CLABSI events in the SICU were Candida species (x4), Enterococcus species (x2), Staphylococcus epidermidis (x3), and Kluyvera ascorbate. Various lines were associated with infection, including (1) triple-lumen, (3) dialysis catheters, (5) PICC, and (1) femoral cooling catheter. 

**Figure 2:**
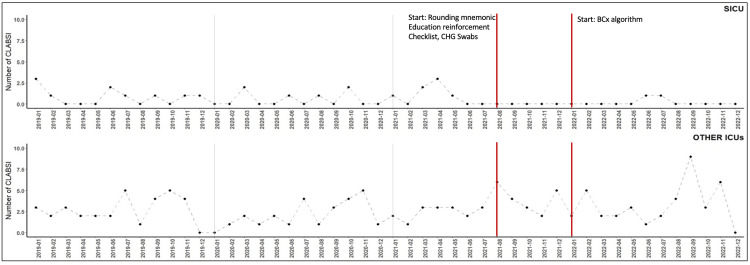
Count of SICU and Non-SICU CLABSI Cases From January 2019 - December 2022 CLABSI: Central Line Associated Blood Stream Infections; SICU: surgical intensive care unit

Figure 2 shows absolute CLABSI events for the SICU and all other ICUs before, during, and after the intervention. While case trends remained stable (without longitudinal decrease) prior to the QI intervention, the SICU showed a sustained reduction of CLABSI at or near zero following the implementation of the bundle in July 2021. This reduction is sustained over 17 months. Compared to the SICU, other health system ICUs during this same period consistently showed greater than or equal to 2 CLABSI events per month.

## Discussion

A team of multidisciplinary stakeholders in the SICU utilized an A3 process to identify potential causes of a high CLABSI rate. Through iterations of the A3 cycle, they created an evidence-based CLABSI reduction bundle associated with a sustained reduction in CLABSI incidence over 17 months. This bundle included changes across multiple aspects of line management, including indication and removal, insertion procedures, daily maintenance tasks, antiseptics, and ordering blood cultures. During this time, other ICUs in the health system (unexposed to the CLABSI reduction QI project) did not experience sustained reductions in CLABSI incidence.

While the various bundle components were generated organically from SICU staff, they resulted in solutions consistent with suggested guidance on preventing catheter-related bloodstream infections (CRBSI). The SICU bundle included elements related to CHG use for skin antisepsis, proper PPE during the procedure, prompt removal of unnecessary catheters, and daily maintenance of indwelling catheters. These changes reflect guidance for key components of CLABSI reduction bundles [13]. For example, CHG is recommended as a first-line agent for skin antisepsis before catheter insertion and has been shown to decrease CRBSI when a solution of 1% or greater concentration is used [13-17]. While there are many studies on CHG’s effectiveness in high concentrations, there is little discussion on the size of the swab used. Larger swabs may have guided staff to disinfect a larger area around the insertion site and thus reduce the likelihood of contamination. 

Various studies also stress the importance of nursing care in preventing CRBSI and supporting using checklists to ensure procedures are done correctly and to empower staff to stop them if needed [13,18-19]. CLABSI bundles as a whole have been shown to reduce CLABSI incidence by upwards of 4 catheter days [20]. Despite this, less than 50% of ICUs in the US have a bundle policy for CLABSI reduction [13]. This allows hospitals and individual ICUs to institute similar bundles to reduce CLABSI and other CRBSI formally. The findings from this QI initiative and these recommendations may provide health systems with additional strategies to prevent CLABSI and provide consistent and evidence-based supportive care to critically ill patients.

There are limitations to this study. QI projects generally have limited control over experimental and control conditions, leaving them open to confounding variables. In this case, only the SICU implemented the bundle, whereas the other health system ICUs did not, creating a control group for what could be considered a natural experiment. Natural experiments can be useful in settings where conditions are not conducive to experimental manipulation [21]. However, creating these conditions in the real world can be difficult. This can complicate determining the direct cause for the reduction in CLABSI incidence. Additionally, because the bundle included a number of changes, it is unclear if an individual or the combination of changes led to the reduction. There may also be patient or ICU-level factors affecting the incidence rate in a particular ICU that are not controlled for during the QI initiative.

## Conclusions

CLABSI is a significant hospital complication that can have deleterious effects on patients in the ICU. By using an A3 framework, members of the SICU were able to create and implement a bundle of changes that successfully reduced the CLABSI occurrences to zero for over 10 consecutive months and no more than 2 events over 17 months. Other ICUs in the health system maintained comparably higher event rates during this time. This bundle may provide additional methods for other ICUs aiming to reduce their CLABSI rates. In addition, similar project designs may lend themselves well to natural experiments allowing comparison of different interventions on outcomes.
